# Beyond Signatures: Leveraging Sensor Fusion for Contextual Handwriting Recognition

**DOI:** 10.3390/s25072290

**Published:** 2025-04-04

**Authors:** Alen Salkanovic, Diego Sušanj, Luka Batistić, Sandi Ljubic

**Affiliations:** 1 University of Rijeka, Faculty of Engineering, Vukovarska 58, HR-51000 Rijeka, Croatia; luka.batistic@riteh.uniri.hr; 2 Center for Artificial Intelligence and Cybersecurity, University of Rijeka, R. Matejcic 2, HR-51000 Rijeka, Croatia; 3 Faculty of Engineering, Juraj Dobrila University of Pula, Alga Negrija 6, HR-52100 Pula, Croatia; diego.susanj@unipu.hr

**Keywords:** authentication, biometrics, digital signatures, handwriting recognition, sensor fusion

## Abstract

This paper deals with biometric identification based on unique patterns and characteristics of an individual’s handwriting, focusing on the dynamic writing process on a touchscreen device. Related work in this domain indicates the dominance of specific research approaches. Namely, in most cases, only the signature is analyzed, verification methods are more prevalent than recognition methods, and the provided solutions are mainly based on using a particular device or specific sensor for collecting biometric data. In this context, our work aims to fill the identified research gap by introducing a new handwriting-based user recognition technique. The proposed approach implements the concept of sensor fusion and does not rely exclusively on signatures for recognition but also includes other forms of handwriting, such as short sentences, words, or individual letters. Additionally, two different ways of handwriting input, using a stylus and a finger, are introduced into the analysis. In order to collect data on the dynamics of handwriting and signing, a specially designed apparatus was used with various sensors integrated into common smart devices, along with additional external sensors and accessories. A total of 60 participants took part in a controlled experiment to form a handwriting biometrics dataset for further analysis. To classify participants’ handwriting, custom architecture CNN models were utilized for feature extraction and classification tasks. The obtained results showed that the proposed handwriting recognition system achieves accuracies of 0.982, 0.927, 0.884, and 0.661 for signatures, words, short sentences, and individual letters, respectively. We further investigated the main effects of the input modality and the train set’s size on the system’s accuracy. Finally, an ablation study was carried out to analyze the impact of individual sensors within the fusion-based setup.

## 1. Introduction

Biometric identification has received considerable attention and is increasingly becoming a standard practice. Systems in this domain are typically divided into two main categories: unimodal and multimodal. A unimodal system utilizes a single trait to identify and recognize users, analyzing unique biological or behavioral characteristics. A multimodal system employs a combination of various biometric traits to enhance both accuracy and security. Combining data from multiple sources can significantly improve the accuracy of such systems [1].

Biometric identification holds a significant role in the domain of person recognition and provides a crucial security measure for preventing a variety of fraudulent activities. Document identification remains heavily reliant on handwritten signatures, which are utilized extensively in the financial, administrative, and other sectors. Numerous research studies focus on handwriting analysis for verification purposes, with such approaches being more widely used than person recognition tasks. The process and movements involved in creating handwritten signatures are unique to each person, making signatures a reliable biometric for identity verification [2]. Signature identification is effective due to the difficulty in replicating these personalized characteristics, which poses a significant challenge for forgers [3]. To address different verification needs, two primary categories are commonly used: offline (static) and online (dynamic) [4].

Offline verification methods have previously attracted particular interest, predominantly relying on images of signatures without additional attributes. A conventional method for acquiring signatures involves scanning various documents, including bank cheques, contracts, invoices, and financial agreements. The system proceeds to detect, extract, and analyze distinctive characteristics from the acquired image. Typical analysis involves examining the geometric characteristics of the signature, such as its shape and size ratios. In offline circumstances, however, verifying signatures is particularly challenging due to the absence of a trace of the signing procedure [5].

As devices equipped with an expanding array of integrated sensors become more widespread, research in this domain has increasingly focused on the field of online signature verification (or identification). These methods facilitate the acquisition of dynamic signature characteristics, a task challenging to achieve solely through conventional pen-and-paper means. Here, the system utilizes a variety of devices and sensors to acquire additional data and extract more features during the signing procedure. The data encompass various aspects such as duration, pressure level, pen movements (up and down), pen tilt, signing velocity, writing trajectory, and stroke order [6]. Together, they provide valuable insights into the intricate dynamics of the signing process. The use of dynamic features notably enhances identification reliability and yields a more effective verification outcome [7].

In contemporary contexts, tablet computers with corresponding styluses are frequently employed for the acquisition of signatures. Moreover, devices like smartphones and smartwatches are becoming more accessible and are equipped with various types of sensors. Inspired by this observation and the findings of others, we initially developed a proof-of-concept apparatus to capture various biometric characteristics during writing on a touch-sensitive display. The potential for using this particular kind of handwriting as a method of identification arises from its connection to several characteristics, including shape, movement, and variation. These attributes exhibit distinct patterns that are unique to each individual, making the signature and the act of signing itself a representation of a person’s identity.

Research presented in this manuscript focuses on a unimodal online solution for person recognition, utilizing a single behavioral attribute, specifically handwriting. It involves an implementation of the sensor fusion concept, distinguishing it from comparable research that mostly depends on a singular type of sensor or device. Our goal was to introduce a larger number of sensor readings that predominantly come from standard smart devices (rather than specifically designed hardware) into the analysis of handwriting dynamics. Also, in contrast to related studies primarily focused on gathering user signatures, our approach involves the collection of diverse handwriting samples, including signatures, short sentences, words, and individual letters. Accordingly, this study investigates the feasibility of recognizing a person through various forms of handwriting, without relying exclusively on signatures. It expands the analysis to general handwriting, as signatures are learned and practiced, while natural handwriting reflects more spontaneous and unique traits [8,9,10]. In addition, data on handwriting dynamics are collected not only for stylus input but also for finger input, which is still the predominant way of interacting with touchscreen devices. In this way, the research was extended to the analysis of the proposed solution with regard to the input modality.

To provide a brief summary, the following contributions are included in this work:

- This paper introduces a sensor-fusion-based method for user identification via touchscreen handwriting, which goes beyond conventional signature-based systems. It incorporates additional handwriting forms for person recognition, namely sentences, words, and individual letters. This investigates whether a person can be successfully identified based on handwriting when the given input pattern is the same for all users.
- A comprehensive dataset is obtained by conducting a controlled experiment with 60 participants, featuring dynamic handwriting data coming from both stylus and finger inputs. The dataset includes details across multiple sensor types, making it a valuable resource for future research in handwriting-based person recognition.
- A machine learning model based on a convolutional neural network (CNN) is designed for both the feature extraction and classification of handwriting. This model achieved high accuracies across different forms of handwriting. Additionally, the accuracy of the model was analyzed with regard to the effect of input modality (stylus vs. finger) and train set size.

The remaining sections of this work are structured as follows: The related work is outlined in Section 2. An overview of the apparatus, experiment procedure, obtained dataset, and CNN model development is provided in Section 3. The research findings, along with the corresponding discussion, are presented within Section 4. Finally, in Section 5, we bring the conclusions and outline for future work.

## 2. Related Work

This section first presents an overview of solutions, methods, and approaches that leverage one or more distinct sensors for signature verification, distinguishing between genuine and forged signatures. It then continues with a review of studies related to person recognition through handwriting.

### 2.1. Handwriting Verification

mmSign [11] and mmHSV [12] represent online handwritten signature verification platforms using commercial mmWave radar to capture in-air hand motions. mmSign focuses on noise reduction, feature extraction, and meta-learning strategies for model adaptation using a custom dataset. mmHSV evaluates robustness against various attack scenarios by combining hand shape and writing process features with custom-collected data. PPGSign [13] uses photoplethysmography (PPG) sensors to capture blood flow changes during hand movements for signature verification. Due to limited access to raw PPG data from commercial devices, the authors developed the PPGSign Band as a proof-of-concept and introduced a proprietary dataset of raw PPG data.

ASSV [14] and SilentSign [15] both use acoustic sensing to capture pen movements during the signing, with device speakers and microphones detecting signal variations. ASSV requires specific phone positioning and distance from the writing surface, while SilentSign offers more flexibility with a larger signature area. SilentSign uses traditional classifiers like logistic naive Bayes (NB), linear regression (LR), random forest (RF), and support vector machines (SVMs), whereas ASSV utilizes deep learning with CNNs for binary classification. Sadak et al. [16] used pen–paper friction sounds for signature verification via mobile device microphones, converting them into features for dynamic time warping (DTW) comparisons of genuine and forged signatures. Like ASSV, this solution requires no external hardware but has limitations with microphone placement and pen type. SonarSign [17] broadcasts inaudible acoustic signals and analyzes their echoes through a channel impulse response (CIR), implementing an attentional multi-modal Siamese network for verification. SVSV [18] combines sound captured via a smartphone’s microphone and vibration data from inertial sensors (accelerometer and gyroscope) for signature verification, using a CNN-based one-class classifier.

The growing popularity of wrist-wearable devices makes them valuable for handwriting verification research. For instance, Ramachandra et al. [19] analyzed writing patterns using smartwatch accelerometer data, processed with a 2D continuous wavelet transform (CWT) and pre-trained ResNet50 features, across two different smartwatches and three writing scenarios. Li and Sato [20] used a Siamese recurrent neural network (RNN) to analyze motion sensor data from smartwatches during signing, finding that combined accelerometer and gyroscope data yield the best results. Similarly, Realme [21] employs a MetaWear sensor with a gyroscope and accelerometer for signature verification on paper.

Multiple works explore in-air signature verification through different approaches. Guo and Sato [22] evaluated CNN architectures for classifying the 3D reconstructions of in-air signatures, with ResNet and Adagrad optimizers yielding the best performance. RNNs have also been successfully utilized to analyze gyroscope and accelerometer data [23,24]. AirSign [25] combines sound and motion sensors, using smartphone components (speakers, microphones, accelerometers, and gyroscopes) to capture hand geometry, signature, and motion data. Atreya et al. [26] developed a method using the 3D pen trajectory and a 2D spatial–temporal CNN, evaluated with the LeapMotionAirSignature [27], SVC-2004 [28], and custom T3AAS-v1 datasets.

Various studies focus on digital pen-based signature verification, using both commercial and custom sensor-embedded stylus systems. Seki [29] employed the Anoto pen system, a digital tool similar to a ballpoint pen, to capture dynamic data through a built-in camera and pressure sensor. Subedi et al. proposed two sensorized stylus systems for signature verification [30,31]. The first uses two accelerometers to classify signatures based on grip, stroke patterns, and speed. The second employs a low-cost, 3D-printed stylus with accelerometers to capture kinematic data, classified by a multilayer perceptron (MLP). Lech and Czyżewski [32] developed a wireless biometric pen with a three-axis accelerometer, gyroscope, surface pressure sensor, and two touch pressure sensors. It mimics a ballpoint pen, with signature verification based on six dynamic measures derived from the DTW method. Zhou et al. [33] combined offline and online verification by collecting physical signatures and digital data with a smart pen equipped with a camera and pressure sensor.

The following paragraphs discuss additional relevant studies focusing on CNN-based approaches for signature verification, exploring various datasets and architectural configurations. Zhang et al. [34] proposed a federated learning framework with 1-D CNN, while Ashwin Shenoy et al. [35] employed CNNs for digital cheque clearance. Singh and Viriri [36] employed CNNs as feature extractors with RNN classifiers, using the publicly available SigComp 2009 [37] and SVC-2004 [28] datasets. The model’s low validation scores were due to the limited sample sizes. Roszczewska and Niewiadomska-Szynkiewicz [38] explored CNN-based mobile signature verification using VGG-16, SigNet, and the lightweight SigNetExt. Trained on the MobiBits dataset [39] with data augmentation, SigNetExt showed strong robustness against skilled forgeries, proving effective for mobile-based verification. Leghari et al. [40] proposed a deep learning approach using CNNs, applying data augmentation and leveraging a deep aggregated convolutional feature representation on a custom-built online signature repository. Zheng et al. [41] employed a depth-wise separable convolutional neural network (DWSCNN) for signature verification, achieving similar performance to traditional CNNs while reducing parameters, resource usage, and execution time, utilizing the MCYT-100 [42] and SVC-2004 datasets. OSVConTramer [43] is a solution with a hybrid architecture combining CNNs and Transformers for online signature verification, evaluated on the MCYT-100, SVC-2004, and SUSIG [44] datasets, focusing on one-shot learning scenarios. Ji et al. [45] introduced a multi-path feature fusion network combining static and dynamic handwriting data for online signature verification, using ResNet18 and a 1-D CNN, evaluated on the BiosecurID [46] and custom CIEHD datasets.

In contrast to deep learning methods that automatically extract features, several studies, including Bensefia et al. [47], employed a grapheme-based verification approach that uses a mutual information criterion and relies on predefined handwriting structures rather than learned representations. These approaches differ from writer identification methods in that they focus on validating a candidate rather than selecting the most likely writer from a set.

### 2.2. Handwriting Identification

Considering person recognition via signature or handwriting, various studies employ CNN models (both pre-trained and custom). Li et al. [48] used a deep CNN with ResNet-18 and a feature pyramid network (FPN) for multi-scale fusion, with the MCTOP algorithm for enhancing the recognition of small signature samples. Çiftçi and Tekin [49] demonstrated the effectiveness of fine-tuning pre-trained CNNs for biometric signature recognition by testing five architectures. Among them, GoogLeNet and Inception-V3 achieved the highest accuracy, EfficientNet-B0 balanced efficiency and accuracy, while MobileNet-V3 Large was ideal for lightweight applications. Pokharel et al. [50] proposed a deep learning approach for signature identification using pre-trained GoogleNet with backpropagation, automating feature extraction to reduce training time. Calik et al. [51] proposed LS2Net, a CNN for signature recognition that improves training with batch normalization and simplifies classification with a class center-based classifier (C3), outperforming models like VGG-16 with limited training data. Culqui-Culqui et al. [52] developed CNN-GC, a CNN algorithm designed for portable devices that use convolutional and pooling layers with ReLU activation, outperforming two baseline CNNs. Rahim et al. [53] developed a CNN-BiLSTM model for identifying individuals through Bengali script handwriting using the novel BHW dataset. The model outperformed standalone CNN and BiLSTM approaches by combining CNNs for spatial and BiLSTM for sequential features. Hasan et al. [54] introduced a hybrid model combining CNN, BiGRU, and BiLSTM layers for person recognition using 10 specific keywords. Poddar et al. [55] proposed a unique integration of CNNs with traditional feature extraction methods (Crest–Trough, Harris Corner Detection, and Speeded-Up Robust Features (SURFs)). Gumusbas and Yildirim [56] proposed capsule networks, outperforming CNNs in person identification tasks, particularly with lower input resolutions and limited data.

As for non-Latin scripts, Rexit et al. [57,58] developed a multilingual offline signature recognition system for Uyghur, Chinese, Kazakh, and Han languages, using feature fusion, principal component analysis (PCA) for dimensionality reduction, and classification with RF and k-nearest neighbors (k-NN). Mo et al. [59] focused on Uyghur and Kyrgyz signatures, classifying fused features using a SVM with a radial basis function kernel. Al-Shamaileh et al. [60] introduced an Arabic handwriting dataset for writer authentication using k-NN with DTW and SVM classifiers, while Dargan et al. [61] applied a similar classification approach for Devanagari writers.

Regarding contactless alternatives to traditional methods, CNN models with modified input representations have been employed to accommodate 3D air signatures captured via leap motion sensors [62]. Similarly, Chuen et al. [63] introduced a contactless signature recognition system using a custom multi-scale CNN architecture with the Microsoft Kinect sensor. Khoh et al. [64] proposed a similar approach with Kinect depth images, offering another contactless solution where SVM classification showed the best results. Ghosh et al. [65] introduced a spatio-temporal Siamese neural network (ST-SNN) for identifying 3D signatures, combining CNNs for spatial and LSTM for temporal features to enhance forgery resistance.

Several studies have explored hybrid approaches that combine feature extraction techniques and machine learning classifiers for handwriting recognition, focusing on both offline and online methods. Kette et al. [66] found that local binary patterns (LBPs) work well for small datasets but struggle with larger ones. Hezil et al. [67] found that combining binary statistical image features (BSIFs) and LBP features, along with overlapping zoning techniques, balances feature complexity and performance. Sriwathsan et al. [68] showed that SURF features outperform the scale-invariant feature transform (SIFT) for signature recognition, achieving better results with SVM and K-means clustering. Inan and Sekeroglu [69] developed a backpropagation neural network (BPNN) with a custom signature database, testing robustness against noise and achieving notable recognition rates with Salt-and-Pepper noise. For online handwriting recognition, Leghari et al. [70] evaluated five machine learning classifiers and found that ensemble methods, such as random forest and extra tree, outperform traditional classifiers in signature identification tasks. Akash et al. [71] and Begum et al. [72] collected real-time handwritten data, extracting six features from predefined keywords and phrases. They evaluated classifiers like SVMs, LR, RF, and linear discriminant analysis (LDA), achieving the highest accuracies with SVMs and LR.

Table 1 summarizes recent person identification studies through handwriting, detailing the different devices or sensors utilized, the handwriting forms examined, and the reported accuracy. The table emphasizes studies that, like ours, focus on person recognition (identification) through handwriting, rather than verification. While these studies typically utilize limited sensor setups centered on signatures, our research employs a broader approach through sensor fusion, capturing diverse biometric characteristics from various handwriting forms using multiple sensors.

### 2.3. Datasets

As far as datasets for handwriting research are concerned, the vast majority of publicly available resources contain only digitized signatures. These datasets usually contain a variety of authentic signatures and forgeries and are predominantly used in signature verification studies. Some of the well-known datasets are described in the following.

The CEDAR Signature dataset [73] is a popular benchmark used primarily for offline signature verification and contains the signatures of 55 people. It contains both genuine signatures and forgeries, with 24 genuine signatures and 24 forgeries available for each writer. The dataset contains only images of signatures, scanned at 300 dpi in grayscale. Pre-processing steps such as binarization, noise removal, and slant normalization were performed to improve image quality.

The GPDS-960 dataset [74] is another offline signature dataset with handwritten signature samples of 960 individuals. A total of 24 genuine signatures were collected from each of the 960 individuals, along with 30 simple forgeries per person created by inexperienced forgers attempting to replicate the genuine signatures. The signatures were digitized with a scanner device in 256 grayscale levels at 300 dpi.

The MCYT-330 signature subcorpus [42] focuses exclusively on signature data, capturing both online and offline modalities. For each of the 330 individuals, 25 genuine signatures and 25 highly skilled forgeries were collected, created by other participants who had practiced mimicking the genuine signature before providing the forged samples. The online data, captured using a Wacom Intuos tablet, include pen position, pressure, azimuth, and tilt, while the offline data consist of images of the written signatures.

The SVC2004 dataset [28] is an online signature collection that contains both authentic signatures and skilled forgeries in English or Chinese. The collection consists of 100 different sets of signatures, each from one contributor. For each individual, there are 40 samples, of which 20 are genuine signatures and 20 are skilled forgeries created by at least 4 other people who practiced imitating the genuine signature before forging it. Similar to the MCYT dataset, the data were captured using a Wacom Intuos tablet. However, access to the dataset is partially restricted as only the training set is publicly available.

The SUSIG dataset [44] is an online signature verification dataset that contains over 3000 genuine and 2000 skilled forgeries in the entire database. The database was compiled in two parts, the visual and blind subcorpora, with 100 different people contributing to each part. The data were captured dynamically using pressure-sensitive tablets. The visual subcorpus used a tablet with an LCD display that provided visual feedback, while the blind subcorpus used a tablet without such feedback. Skilled forgeries were obtained after the forgers practiced by watching a playback of the genuine signature creation.

The BiosecurID dataset [46] is a multimodal biometric database that contains both offline and online handwritten text and signatures from 400 participants. It contains lowercase Spanish text, uppercase words and digits, and signatures that are both genuine and skillfully forged. Each participant provided sixteen genuine signatures and twelve skilled forgeries in four sessions. The other handwriting tasks (text, words, and digits) included only genuine samples, with four of each type being collected per participant. Data for both the signatures and general handwriting were captured using a Wacom Intuos tablet and an ink pen, which allowed simultaneous collection of online dynamic signals and offline ink-on-paper versions, which were then scanned. Consequently, the database contains both the dynamic, temporally sequenced data (online) and the corresponding static scanned images (offline).

A summary of the previously described datasets can be found in Table 2. It can be seen that most of the known datasets focus exclusively on signatures and not on other forms of handwriting. When online data are available, it is mostly from graphics tablets, so the concept of sensor fusion, which encompasses a broader range of dynamic data, is often neglected. This also leads to the fact that none of the datasets include finger-based data, although finger input is still the dominant way of interacting with modern touchscreens. As mentioned above, most of the studies using these datasets focus on verifying signatures in a binary classification where the goal is to distinguish between genuine and forged signatures. Therefore, these datasets may not be entirely suitable for testing our models in a comparative analysis with other approaches. Namely, such a comparison might only refer to the context of signatures and completely disregard other forms of handwriting and different input modalities. Investigating this context beyond signatures is one of our goals, and the next section looks in detail at the identified research gaps that are the focus of our study.

### 2.4. Research Gap

Most of the reviewed works in the field of handwriting biometrics are centered on verification systems, which involve distinguishing between genuine and forged handwriting samples. Such systems represent a two-class classification problem, where a decision is made on whether a sample is authentic or forged. While this is a crucial aspect of biometric security, these studies often overlook the broader context of user recognition across various handwriting forms. Our study addresses this research gap by broadening the focus from verification to a wider range of handwriting identification tasks, a general process of detecting the handwriting owner that involves multi-class classification. Specifically, our approach extends beyond signatures for handwriting-based person recognition to include various handwriting forms, such as sentences, words, and letters. We thereby extend the research by investigating whether a person can be successfully identified based on handwriting when the given input is the same for all users. This expansion has resulted in the introduction of a newly curated dataset, consisting of measurements from different types of sensors during writing with both a stylus and a finger.

Furthermore, many of the reviewed studies rely on specialized hardware, such as custom-designed pens or complex sensor systems, which are often limited and costly. In contrast, our approach utilizes readily available off-the-shelf components and standard smart devices. This not only simplifies the experimental process but also enhances the reproducibility of the findings. Unlike most existing solutions that are mainly based on the use of a single sensor (or a rather small number of sensors), our approach implements a sensor fusion concept, intending to collect and utilize a much larger amount of handwriting dynamics information.

The proposed handwriting-based biometric system has several potential practical applications in secure authentication and user recognition. One potential application is digital identity identification in banking and financial transactions, where handwriting dynamics can provide an additional layer of security against fraud. Another potential use case is access control and device security, where handwriting recognition can serve as an alternative biometric authentication method for unlocking smartphones, tablets, or secure workstations.

This system offers an alternative or supplement to traditional authentication methods such as passwords, PINs, or fingerprint scanning. For comparison, fingerprint systems can be compromised using latent fingerprint residues left on sensors or by artificial fingerprint replication. In contrast, handwriting recognition systems analyze dynamic behavioral traits such as writing speed, pressure, and stroke order, which are more difficult to replicate or forge. Additionally, handwriting-based recognition does not suffer from the challenges associated with fingerprint wear and tear. Individuals with worn fingerprints due to aging, manual labor, or skin conditions often experience difficulty with fingerprint scanners. Since handwriting is a learned and practiced behavior, it remains relatively stable over time, providing a more consistent identification method for such users.

While signatures have traditionally been used for identifying individuals, for example in various legal and administrative systems, they usually consist of brief, well-practiced movements. This makes them particularly susceptible to forgery, especially when attackers deliberately train to replicate them. While each individual’s signature is unique, analyzing different forms of handwriting, such as sentences, provides greater insight into a person’s dynamic writing characteristics. Additionally, signatures are commonly found on paper or scanned documents, making them more accessible to attackers. In contrast, longer handwriting samples, like complete sentences or words, are less readily available and more difficult for an attacker to obtain. This supports the use of various handwriting types instead of signatures, as employing other handwriting forms as a “signature pattern” would make forgery attempts more challenging by requiring notably more effort to produce a convincing imitation.

## 3. Materials and Methods

### 3.1. Apparatus Description

As described in our previous work, the experimental apparatus within the proposed solution features a variety of sensors from different devices [75]. Specifically, it includes the touch sensor and magnetometer from the tablet, the accelerometer and gyroscope from the smartwatch, the camera from the smartphone, and two external piezoelectric sensors. The components were securely mounted on a perforated 120 cm × 60 cm plate using screws, brackets, and holders to ensure consistent positioning during the experiment.

**Figure 1:**
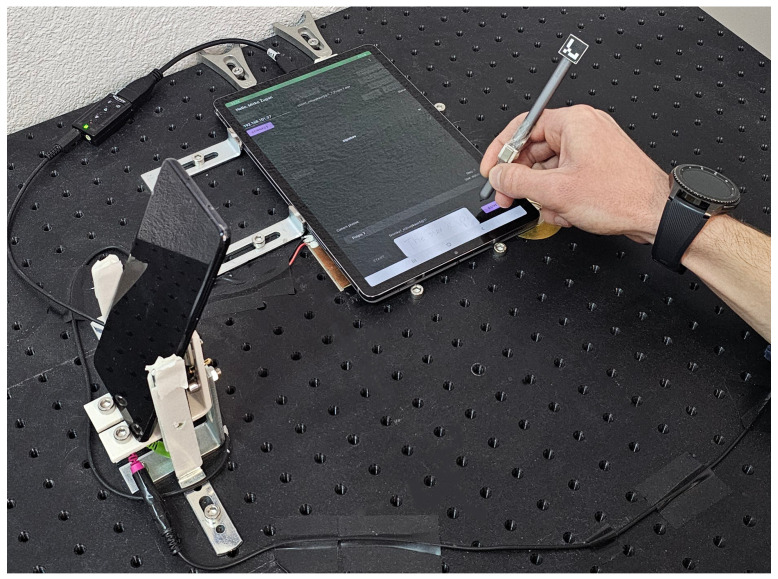
Conducting a controlled experiment using the proposed apparatus. The components comprise a tablet, smartphone, and smartwatch, along with two external piezoelectric sensors.

Figure 1 shows an experiment setup wherein a participant, wearing a smartwatch, uses a stylus on a touch-sensitive screen. Two external piezoelectric sensors are located beneath the tablet—one connected to the tablet and the other to the smartphone. Applications for Android and Tizen OS are developed in order to streamline device synchronization and ensure reliable data acquisition.

The primary component of the apparatus is the Samsung Galaxy Tab S6 tablet, featuring a 10.5″ touchscreen suitable for handwriting input via stylus or finger. The device includes the S Pen stylus, capable of detecting 4096 pressure levels based on the applied force while writing. Due to the constraints of the S Pen Remote SDK, tilt data can not be provided by default.

The tablet incorporates various sensors, and the magnetometer is one of those that is rarely used in similar research. That is why we included permanent magnets in the apparatus. Namely, a pair of neodymium magnets, each with a remanence value of 1.19 T, is utilized to disrupt the magnetic field around the tablet device when writing with a stylus or finger, thus creating a unique magnetic trail. Custom-designed 3D-printed holders secure the magnets, including a ring-shaped holder for finger-writing and a specialized holder for the stylus. Additionally, 3D-printed mounts were created for ArUco markers, square patterns that cameras detect to provide precise 3D position data [76]. An ArUco marker was placed at the top of the stylus to acquire tilt data during writing. The output includes the X and Y coordinates of the ArUco marker readings obtained from videos. From this data, rotation and translation vectors can be obtained to determine the angle at which the user holds the stylus.

Regarding the tablet touch sensor, it operates under display featuring a resolution of 1600×2560 pixels and a density of approximately 287 pixels per inch (ppi). It can detect inputs from both the stylus and the user’s finger, collecting various data during writing, including pressure, size, velocity, rotation, and the precise X and Y coordinates corresponding to each touch event.

When it comes to external sensory components, the tablet was equipped with a piezoelectric sensor, acting as a contact microphone, to collect more data on the pressure exerted on the screen during writing. It captures acoustic oscillations generated by the movement of the stylus or finger, providing information on sliding actions, touches, and pressure applied to the screen or the sensor itself. The audio signal is obtained through the tablet’s microphone input using an external USB 2.0 sound card and a USB-C to USB-A adapter, as the tablet lacks a physical 3.5 mm audio connector.

The experimental setup additionally includes a Samsung Galaxy S9+ smartphone, which was at our disposal. This device was essential because the tablet was unable to establish a connection with the smartwatch due to restrictions imposed by the manufacturer. In contrast, the smartphone enables communication with the smartwatch, allowing for the acquisition of gyroscope and accelerometer data. Thus, the smartphone plays a critical role in facilitating this connectivity. Furthermore, the smartphone’s camera sensor captures the handwriting process and allows for extracting stylus tilt data from the recorded video and ArUco marker tracking.

In addition to using the camera sensor, the smartphone complements touchscreen pressure acquisition with a second, smaller piezoelectric sensor placed beneath the tablet. This sensor is positioned on the opposite side of the first piezo sensor. The advantage of this smartphone model lies in its provision of a physical 3.5 mm audio connector, a feature lacking in the majority of contemporary devices. Consequently, it was considered appropriate to utilize this feature for the connection of an additional piezoelectric sensor of different shapes, dimensions, and characteristics.

Finally, the Samsung Gear S3 Frontier smartwatch was integrated into the apparatus to collect additional data from its built-in sensors. It features an accelerometer and a gyroscope, used to capture the unique dynamics of a user’s writing style. The purpose of the smartwatch is to acquire the motion-based biometric details of the user while they are actively engaged in writing on the tablet screen. Participants were required to wear a smartwatch device on their dominant hand as part of the experimental protocol, ensuring consistency in the data collection process.

### 3.2. Experiment Procedure

Prior to the initiation of the experiment, all participants were asked to sign an informed consent form. Each participant was given the opportunity to examine a document detailing a concise overview of the experiment, its objectives, the data to be collected, and the research goals. Afterward, the participants were engaged in the system familiarization process to understand its operational procedures. Data collection was not initiated during this phase. Following their familiarity with the apparatus, participants were presented with detailed explanations of all experiment methods.

In the beginning, participants were asked to position the smartwatch on their dominant wrist. They were asked to write on the tablet screen utilizing a stylus initially and then using their index finger. During finger-writing, participants wore a custom 3D-printed ring fitted with a neodymium magnet on their index finger. Its purpose was to interfere with the magnetic field values captured by the magnetometer. To ensure accurate measurements, the magnetometer was calibrated at the start of each trial to account for environmental noise.

Considering that handwritten signatures represent a pre-learned action for each individual, this study explores the possibility of user recognition through alternative handwriting forms. The objective is to examine the extent to which, besides signatures, the process of writing sentences, words, or letters varies from person to person, as well as its level of uniqueness for each individual. Hence, aside from obtaining handwritten signatures, the process of collecting data from participants required the writing of short sentences, words, and individual letters.

In greater detail, each respondent was tasked with reproducing their signature 25 times. Regarding the composition of sentences, they pertained to 10 sentences in total. They were primarily composed of a few short words, deliberately selected to encompass all of the letters in the alphabet. Participants were instructed to write each of the sentences a total of 5 times.

Furthermore, 10 words were selected following a methodology similar to the previously described. In this instance as well, each word was required to be written 5 times. Concerning the letters, all characters from the English alphabet were included. In addition to uppercase letters, lowercase letters were also taken into account. In contrast to sentences and words, each letter was written out 3 times.

Each handwriting form (signature, sentence, word, and letter) was collected using two input modalities: first with a stylus and then with a finger. Participants had a designated writing space on the tablet screen, varying in size based on the handwriting form currently being obtained. Throughout the process, the application displayed textual labels to indicate the current sentence, word, or letter being collected, ensuring accurate input. To begin data collection, participants activated a *start* button, which activated all sensors within the fusion-based setup. After finishing the writing activity, selecting the *stop* button halted the sensor readings and stored the data. This method ensured data collection occurred only between these two events, allowing participants to take breaks as needed. The average duration of the experiment for each participant was between 70 and 90 min, including breaks.

In a controlled environment, 60 participants, 12 females, and 48 males took part in the trial. Most were young individuals aged 20 to 24, primarily students, who reported regular use of modern touchscreen devices. Notably, 9 individuals were left-handed. While a more diverse participant pool in terms of age, profession, and cultural background would improve generalizability, such an expansion would require greater organizational and financial resources, as well as considerably more time. This remains a limitation of the study and will be addressed in future research. The experiment and data collection were approved by the Ethics Committee of the University of Rijeka, Faculty of Engineering (approval no. 640-08/23-01/3). Due to privacy concerns, and following the contemporary practices regarding biometric datasets, it was decided in advance that the obtained dataset would not be publicly available for uncontrolled download. Instead, it could made available upon request, subject to a signed confidentiality agreement to ensure the protection of participant data. This agreement should specify the terms and conditions for data usage, so the dataset could be securely shared with researchers who met these requirements.

### 3.3. Dataset

The obtained dataset comprised data collected from the various sensors described in previous chapters during the handwriting process using either a finger or a stylus. The acquired measurements were represented in multiple formats due to the use of different sensor types.

Regarding the tablet device, the touchscreen sensor collected diverse information using the MotionEvent object in Android to report stylus or finger movement events. This included data such as touch position, contact area size and orientation, pressure level, and touch event (start of touch, end of touch, and movement during touch). Velocity data were obtained through the Velocity Tracker API, allowing for the measurement of writing direction and velocity of movement along the X and Y axes. For each touchscreen interaction event, supplementary information, such as the timestamp of the event, was recorded and stored. The Javascript Object Notation (JSON) format was used for storing the aforementioned data, particularly for structuring data relevant to touch events.

A screenshot was taken at the conclusion of each writing session, specifically capturing the handwriting produced by the user. The image of the input was saved in the Portable Network Graphics (PNG) format, ensuring clarity and quality preservation for future reference and analysis.

Within this framework, it was essential to ensure that the sampling rate of the additional sensors matched that of the touchscreen, as it had the lowest sampling rate. Readings from other sensors were considered only when synchronized with the touch event. For example, magnetic field readings (along the X, Y, and Z axes) from the tablet’s built-in magnetometer were stored in the JSON format only when relevant touchscreen input was detected. Although the magnetometer produced significantly higher read rates than the touchscreen, only data pertinent to the specific touch event during writing were recorded. This principle was applied to all other sensors in the apparatus.

Two piezoelectric sensors were positioned beneath the tablet, each on opposite edges, to collect vibrations and pressure changes. Functioning as audio capture devices like standard microphones, the information was stored as separate audio files in the waveform audio file format (WAVE, or WAV). The captured signal contained details regarding gliding, tapping, and applying pressure to the touchscreen, thereby synchronizing with the recorded handwriting data.

Concerning the smartwatch, the integrated accelerometer was utilized to measure acceleration forces, while the gyroscope captured the rate of rotation around all three axes. In this scenario, commands signaling the initiation and termination of sensor recording were relayed from the smartphone to the smartwatch. Consequently, the data were continuously acquired from the very beginning until the conclusion of the writing process. The readings were captured as distinct textual files in the internal memory of the device and later transferred to a computer using our custom script.

Regarding the smartphone device, its camera sensor captures the stylus movement during the handwriting process. Due to the limitations of the S Pen Remote SDK, tilt data could not be obtained, so recorded videos served this purpose. The recordings were trimmed according to the exact timestamps written in a separate text file, based on precise moments at the start and conclusion of the writing process. The outcome of the mentioned process consisted of MP4 video assets corresponding to individual instances of written signatures, sentences, words, or letters. These files were further utilized for ArUco marker detection using a custom Python 3.11 script. Finally, the 3D translation and rotation vectors of the detected markers were separately stored in text files to determine the stylus’s inclination.

The obtained dataset is divided into train and test sets, where the test set is further split into gallery and query subsets. The train set size represents all biometric samples (recordings) from 10, 20, or 30 randomly selected participants. The size of the test set, comprising the gallery and query subsets, depends on the train set, as it is derived from the remaining participants (50, 40, or 30, respectively). Depending on the input modality (stylus or finger), handwriting form (signature, sentence, word, letter), and train size, 2×4×3 datasets were generated and observed independently. For the sentence and word handwriting forms, the gallery subset comprised the second and fourth samples, while the query subset contained the first, third, and fifth samples. For the letter handwriting form, the second sample was included in the gallery subset, while the first and third samples were assigned to the query subset. Since there were 25 samples for signatures, the assignment to gallery and query subsets was determined using their ordinal positions after applying a modulo operation with the value 5. This facilitated the same selection pattern applied to the sentence and word samples, ensuring a consistent and systematic distribution across the gallery and query subsets.

The sensor data were organized into 24 distinct vectors, each containing specific measurements from a sensor during handwriting sessions. As an illustration, readings from the magnetometer along the X, Y, and Z axes formed three vectors, while six vectors were created from the translation and rotation values of ArUco markers along the same axes.

The analysis of the effects of individual sensor measurements on model performance is a challenging task due to the exponentially increasing time required to train all models. Namely, treating each individual data vector as an independent variable would require training the models with an impractically large number of vector combinations ($2^{24}-1$), complicating both computational processing and interpretation of the results. To address this, we categorized the 24 vectors into 6 different sensor subsets based on the type of data and the sensors used to acquire them. This categorization simplifies the analysis and ensures that it remains computationally feasible. When grouping the data vectors into subsets, we took into account the context dependency of the available measurements. For example, two piezoelectric sensors together provide insight into the pressure levels during writing, while the combination of an accelerometer and a gyroscope (with a total of 6 measurements) provides a more comprehensive understanding of the dynamics of hand movement. Each category includes specific measurement parameters as follows:

- **Touchscreen**: Includes touch positions (X, Y) and touch velocities (in X and Y directions) while writing.
- **Magnetometer**: Measurements of magnetic field along the X, Y, and Z axes.
- **Input specific**: Tracks stylus tilt, stylus pressure, and finger touch size.
- **Piezos**: Obtained piezoelectric data from two piezoelectric sensors connected to tablet and smartphone.
- **Smartwatch**: Gathers rotational data and acceleration data across three axes from gyroscope and accelerometer.
- **Visual tracking**: Monitors the translation and rotation of a stylus using an ArUco marker.

### 3.4. Data Preprocessing

Handwriting samples were collected while users wrote on the tablet screen, represented as a series of data points. Since multiple sensors are utilized, each data point corresponds to measurements from different internal and external sensors collected during the writing process. Consequently, every point within the handwriting sample has associated sensor readings that capture a range of writing characteristics, including pressure, velocity, magnetometer readings, gyroscope data, and accelerometer measurements, among others. As various sensors typically record at different frequencies, the vectors contain raw sensor measurements that vary in size. For that reason, the length of all vectors (number of data points) is determined by the number of feature points collected by specific sensors during the writing process. Resampling, normalization, and padding address the issues of varying sample sizes and scales.

The initial data preprocessing step involved loading data for the 24 different vectors in the dataset. The length of data (i.e., the number of samples) for each vector varied due to factors like the writing speed,  complexity of the stroke, or hardware sampling rate. Therefore, the next action was to resample the vectors to ensure that each one has a consistent number of samples, denoted as $N_{s}$. This adjustment simplifies the process of feeding the data into deep learning models later. The value of $N_{s}$ is determined by selecting the minimum number of samples obtained from all sensors. In this specific context, it corresponds to the quantity acquired from the tablet’s touchscreen, which recorded the fewest samples. Consequently, the lengths of each feature vector were either reduced or increased to align with $N_{s}$. This adjustment guarantees that all 24 feature vectors are aligned in terms of sample size, allowing for direct comparison and utilization in deep learning models. The maximum lengths of vectors per collected handwriting form are as follows: signature 613, sentence 777, word 607, and letter 130. Based on the number of samples $N_{s}$ in the dataset, a predefined maximum size (MAX_SIZE) of 1024 samples was selected, resizing each feature vector to the same number of data points.

Since different sensors generate data in varying ranges, vectors were normalized to standardize the values. Min-max normalization of the data was conducted, where minimum and maximum values were specified for each vector, according to:

(1) $$\mathrm{vector}\left[i\right]=\frac{\mathrm{vector}[i]-\min(\mathrm{vector})}{\max(\mathrm{vector})-\min(\mathrm{vector})},\forall i\in \left[0,N_{s}\right].$$

In the last phase, right padding is applied to ensure all vectors have the same size, with a target length of 1024 (MAX_SIZE) in this case. This involves expanding the vectors by adding zeros to the right side of the samples until the maximum size is reached.

### 3.5. Feature Extraction

**Figure 2:**
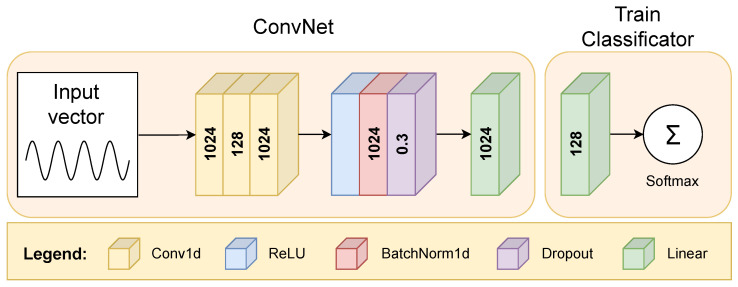
The configuration of a CNN was specifically designed for the objective of feature extraction, accompanied by a training classifier.

The training process for feature extraction entails focusing on the train subset to extract pertinent features. For each of the specified 24 vectors, a distinct feature extractor is trained. Each of the 24 input vectors, corresponding to data from distinct sensors such as the accelerometer, gyroscope, and magnetometer, is processed by a dedicated Conv1D-based feature extractor, which learns local patterns in the data through convolutional layers. As illustrated in Figure 2, the structure consists of three convolutional layers, each with a *kernel size* of 3 and with *stride* and *padding* set to 1.

The first convolutional layer processes the entire input vector of 1024 data points, applying 128 convolutional filters (feature maps). To maintain the original input size of 1024 points, zeros were added at both ends of the vector, preserving local patterns and preventing dimensionality reduction. This enables the first layer to produce 128 distinct feature maps, each capturing specific handwriting patterns. In the second convolutional layer, the network applies another set of 128 filters to the feature maps from the first layer. Rather than processing the raw input data, this layer refines the previously learned feature representations, combining them into more complex representations that capture the relationships between different handwriting aspects.

The choice of three layers for the network was based on empirical testing, where we found that two layers resulted in lower accuracy, while adding more layers did not result in significant improvements. A similar approach was used for selecting the number of hidden nodes, where we found a balance between computational efficiency and classification accuracy through experimentation.

Once the convolutional layers have extracted the relevant features, the rectified linear unit (ReLU) activation function is applied to introduce non-linearity into the model. The batch normalization layer (BatchNorm1d) after ReLU helps the network train faster, minimizes the risk of overfitting, and enhances performance by allowing for higher learning rates [77]. The dropout layer with a probability of 0.3 was implemented following the convolutional layers, ReLU activations, and batch normalization, just prior to the fully connected layers. Dropout contributes to avoiding overfitting to the limited train samples. This technique ensures that the model generalizes better when evaluating new, unseen handwriting samples [78]. The dropout rate of 0.3 was determined by empirical evaluation. Given the sufficiently large size of our dataset, this dropout rate was effective in preventing overfitting while ensuring high model performance. Finally, there is a fully connected linear layer that reduces the number of features from 1024 to 128.

In the training phase, a simple classifier was utilized with a single fully connected linear layer, whose output size corresponds to the train set size. This linear layer was followed by a softmax layer that converts a vector of arbitrary real values into a probability distribution, enabling the classification of handwriting samples. Given that our task is a multi-class classification problem, we used the cross-entropy loss function, which is a widely used solution for such scenarios. It was used together with the adaptive moment estimation (Adam) optimizer to effectively classify handwriting, minimize classification errors, and optimize model parameters [79]. It provides robust adaptive learning rate properties and is therefore suitable for the efficient training of deep networks. The values of other hyperparameters were determined by empirical fine-tuning, with the *learning rate* set to $10^{-4}$ and the *weight decay* set to $10^{-5}$. The training was performed for 1000 epochs with a *batch size* of 32. To facilitate the training process, the Nvidia RTX 4090 GPU with 24 GB VRAM was utilized.

In choosing 1D-CNN over alternative DL-based feature generators, we also considered architectures such as ResNet50, VGGNet, EfficientNet, and DenseNet, where all vectors are combined into 2D images. Ultimately, however, we opted for a 1D-CNN architecture as it offers several key advantages:

- Considering all 24 vectors, the model trains about 5 million parameters, compared to about 25.6 million for models such as ResNet50.
- The memory footprint of the model is only 39 MB, offering a favorable trade-off between size and accuracy.
- Despite its reduced complexity, the model achieves an accuracy comparable to larger and more advanced architectures.

Exploring alternative models, such as RNNs, transformers, autoencoders, or hybrid architectures, could provide further insight into their effectiveness in handwriting recognition. However, the decision to focus on CNNs was motivated by their strong ability to extract locally correlated spatial features, while maintaining a favorable balance between accuracy, computational complexity, and model size. This is particularly effective when dealing with 1D vectors, as in our case, where CNNs can effectively capture changes in handwriting dynamics, such as writing speed or direction, and other associated patterns. While other methods may potentially achieve higher recognition accuracy, this often comes at the cost of higher computational complexity and model size. Another reason in favor of our approach can be found in related work, where many existing studies have successfully used CNN-based solutions for handwriting biometrics.

### 3.6. Classification

Following the training of the feature extractors, we proceeded to freeze all layers of each respective feature extractor. By employing this approach, the weights within the feature extractor network remain unchanged during the classifier training process. This method ensured that the classifier’s training process only modifies its own weights without affecting the previously learned representations of the FEs. For training the classifier, we utilize the gallery subset and subsequently assess its performance on the query subset.

Regarding classifier models, the feature vectors derived from each of the *N* feature extractors are concatenated vertically to form a matrix with dimensions of N×128. The matrix in question consists of *N* rows, each pertaining to an individual feature extractor, and 128 columns designated for every feature vector sample. In this context, the number *N* depends on the number of sensors considered; when all sensors are taken into account, *N* equals 24. As each feature vector contains 128 elements and there are 24 vectors from each sensor, the resulting matrix had a size of 24×128. Thus, each row represents a feature vector from a distinct sensor, and each column corresponds to individual features of these vectors. This matrix served as input to the classifier, which aimed to identify the handwriting owner based on these stacked feature representations. As part of the ablation study, different configurations of feature vectors were used (i.e., using fewer sensor measurements), leading to variations in the matrix size.

**Figure 3:**
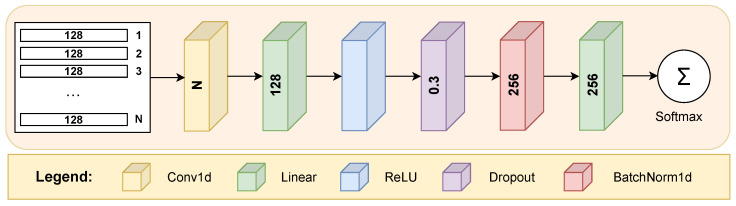
The structural arrangement of the classifier model undergoing training on the gallery subset and subsequently assessed on a designated query subset.

Figure 3 illustrates the classifier model employed within the scope of our research. The architecture starts with a 1D convolutional layer that applies a convolution operation to the input matrix of stacked feature vectors with dimensions N×128, converting it into an output vector of size 1×128. By utilizing a *kernel size* of 3, with both the *stride* and *padding* set to 1, this layer captures local dependencies and patterns within the feature data.

Following the convolutional layer, a fully connected layer helps expand the learned features into a higher-dimensional space. Specifically, this linear layer maps the output from the convolutional layer (size 128) to a new representation with 256 dimensions. The linear layer is sequentially followed by a ReLU activation layer, a dropout layer with a probability of 0.3, and a batch normalization layer. The architecture proceeds with a final linear layer that reduces the 256-dimensional output to match the number of unique classes (number of users in the gallery and query subsets), mapping the learned feature representation to class scores. Finally, the softmax activation function transforms the raw outputs from the last layer into a probability distribution for the class labels.

## 4. Results and Discussion

To address the inherently non-deterministic nature of neural networks and their training process, both the feature extractor and classifier were trained and evaluated across 30 repeated runs. The recorded accuracies from these 30 runs were then averaged, and their standard deviation was calculated.

**Figure 4:**
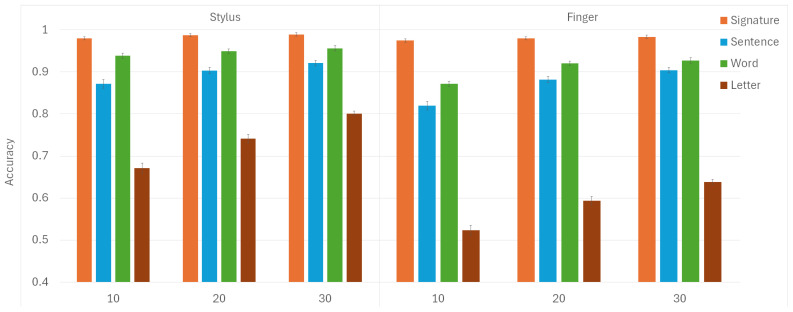
The mean values and standard deviations of classification accuracies for two input modalities (stylus and finger) are presented for three train set sizes (10, 20, and 30) and four collected handwriting forms (signature, sentence, word, and letter).

The mean values and standard deviations for model accuracies are depicted in Figure 4. The highest scores were predictably achieved for signatures, whether written with a stylus or a finger, even when using the smallest train set size. This can be attributed to the unique and distinctive characteristics of signatures as a form of handwriting [80,81,82]. The repeated strokes and patterns in signatures help the model learn and identify these unique features more effectively. On the other hand, all participants wrote the same sentences, words, and letters. Since each person’s signature was unique, creating noticeable inter-person differences, this explains why accuracies were highest specifically for signatures.

In all instances, higher accuracies were attained when participants wrote words as opposed to sentences. Although writing sentences provides more dynamic information than words, intra-person variability in sentence writing introduces noise, making it harder to distinguish between individuals. This could be attributed to possible variations in spacing between words in a sentence, a variability absent when only single words are written exclusively. Since writing sentences takes longer and requires more space, it is reasonable to expect some variation between different attempts by the same participant. When focusing on words, people could experience less cognitive load [83], allowing them to concentrate better on accurately forming each word. Additionally, writing shorter words repetitively could lead to better consistency in execution compared to writing longer, more complex sentences that might require greater mental processing and planning [84].

The lowest accuracies were observed in relation to letters across both input modalities. This can be attributed to how writing individual letters takes noticeably less time than writing sentences and words, resulting in a smaller amount of data collected for analysis. The limited information collected when writing individual letters makes distinguishing between individuals more difficult. With less data, finding enough distinguishing features for the model becomes challenging, especially with the less precise finger input.

The increased accuracy of using a stylus over a finger can be attributed to the greater precision and control it provides. A stylus enables finer motor control, leading to more accurate and consistent writing [85]. Writing with a pen-like tool feels more natural for many individuals, as it simulates the experience of writing on paper. Additionally, using a stylus can reduce screen smudging and enhance visibility, which can be an issue when writing with fingers. The lower accuracy associated with finger input can be attributed to the reduced precision inherent in using a finger on a touchscreen (the well-known fat-finger syndrome). In contrast to a stylus, fingers are wider and less stable, which results in less consistent and more variable input [86].

### 4.1. Main Effects

A three-way (2×3×4) repeated measures ANOVA was employed to investigate the effects of the following within-subjects factors on the model’s accuracy: input modality (stylus, finger), train set size (10,20,30), and collected handwriting forms (signatures, short sentences, words, and individual letters). The Greenhouse–Geisser adjustment (ϵ) was applied in cases where violations of sphericity had been observed. In cases where a statistically significant effect was found, post hoc pairwise comparisons were performed using the Bonferroni correction.

Mauchly’s test indicated that the assumption of sphericity had been violated solely for the variable related to the collected handwriting form: $\chi^{2}\left(5\right)=23.866,p<0.001$.

The results of the RM ANOVA test revealed significant main effects as follows:

1. **Input modality**—The model’s mean accuracy differs statistically significantly between two input modalities: F(1,29)=10915.991, p<0.001, $\eta^{2}=0.997.$
2. **Train set size**—The size of the train set significantly affects the accuracy of the model: F(2,58)=3615.583, p<0.001, $\eta^{2}=0.992$.
3. **Handwriting form**—Significant main effect was observed for collected the handwriting form as well: F(2.193,63.609)=38213.282, p<0.001, ϵ=0.731, $\eta^{2}=0.999$.

Hence, regarding input modality, stylus-based handwriting results in a higher model performance (0.892±0.103) when compared to finger-based input (0.835±0.16), with a statistically significant difference (p<0.001). Writing with a stylus seems to result in more consistent handwriting strokes and patterns across repeated writing attempts. In contrast, finger-writing on a touchscreen appears to introduce greater variability, possibly due to less precision and control over the writing movements.

When it comes to the train set size, post hoc analysis revealed that the accuracy of the model increases significantly with the larger train set size. Specifically, the model proved to be significantly more accurate when a train set size of 30 was utilized (0.89±0.118), compared to cases with a train set size of 20 (0.87±0.136) and 10 (0.831±0.16)—with p<0.001 for both pairwise comparisons. The difference in accuracy with regard to the train set sizes of 10 and 20 also proved to be statistically significant (p<0.001). This aligns with initial expectations, as a larger dataset helps mitigate overfitting by preventing the model from memorizing specific examples in the train set.

With regard to the collected handwriting form, the model exhibits the best accuracy when signatures are employed (0.982±0.005), followed by other handwriting forms in this particular order: words (0.927±0.03), sentences (0.884±0.036), and individual letters (0.661±0.1). Post hoc analysis revealed a significant difference in all pairwise comparisons observed herein (with p<0.001 in all cases).

In terms of comparing with other studies, Table 1 in Section 2 presents a list of relevant studies in this domain. Instead of focusing on verification systems like most studies in the related work, the table highlights research that, like ours, identifies individuals through handwriting. Alongside the compilation of utilized sensors, the table displays the handwriting forms used for user recognition, as well as the reported accuracy from the related studies. The comparison might be more meaningful when limited to studies involving online (dynamic) handwriting, specifically those using smartphones or tablets. In relation to similar research, our solution justifies the introduction of a greater number of sensors for person recognition via handwriting. The proposed solution has attained high accuracy for signature recognition (0.982), which is adequately comparable to the other studies examined. In addition, it has demonstrated high accuracy levels in recognizing words (0.927) and short sentences (0.884), with a lower accuracy for individual letters (0.661). The obtained results support the idea of utilizing various handwriting forms, thus going beyond signatures only, as possible alternatives for user identification. We also provide an analysis in relation to the input modality by comparing handwriting recognition with regard to input with a stylus or a finger. This approach enables a more comprehensive assessment of an individual’s writing style on a touchscreen device.

### 4.2. Ablation Study

The aim of the ablation study is to examine the impact of each of the six sensor subsets on the accuracy of handwriting-based person recognition. This process involves removing one sensor subset at a time to assess its influence on the accuracy of the model with the remaining subsets. For instance, if the exclusion of a particular sensor subset results in an increase in model accuracy, it suggests that this subset has a negative impact on user identification in the observed context. Hence, the model training is additionally conducted for six setups independently, each of which had five active sensor subsets. Results concerning the attained accuracies are presented in Table 3. In the respective procedure, the train set size was fixed to 30, as this set size was shown to produce the highest accuracies. Obtained accuracies are provided for different input modalities (stylus or finger) and distinct handwriting forms (signatures, sentences, words, and letters). Each row corresponds to scenarios in which a specific sensor subset was disabled, except for the last row, which includes results when all subsets within the fusion were active. From the table data, it is evident that the exclusion of certain sensor subsets impacts accuracy differently across the observed categories.

For recognizing a signature entered with a stylus, the model achieves its highest accuracy (0.9911) when either the touchscreen or piezo sensors are excluded. This outcome suggests that these sensors do not enhance signature recognition when using a stylus, possibly due to the characteristics of signature dynamics, which involve a pre-learned action. In contrast, when finger input is employed, the model reaches peak accuracy (0.9911) when the piezo sensor is excluded, indicating its negative impact on accuracy in this scenario. When all sensors are included in the fusion, the model’s accuracy slightly decreases for both stylus (0.9866) and finger (0.9844) inputs, highlighting that the touchscreen and piezo sensors may introduce noise or redundancy rather than improve recognition. Regarding the impact of magnetic field measurements, we can see that the exclusion of the magnetometer results in a slight decrease in accuracy for both stylus and finger inputs. This indicates that the magnetometer captures distinct patterns during the signing process, irrespective of whether a permanent magnet is mounted on the stylus or the finger during writing.

In stylus-based sentence writing tasks, the model achieves its best accuracy (0.9263) when the full fusion setup is active. This outcome suggests that a multi-sensor approach contributes valuable information for sentence recognition with stylus input, which involves more complex and extended strokes compared to signatures. Conversely, when relying on finger input data only, the model records its highest accuracy (0.9163) when input-specific sensors are excluded. The reason for this may be the lack of data in the *input-specific* sensor subset when writing with a finger. Specifically, while stylus input involves data on tilt and pressure level, these are not available for finger input and are substituted with zeros. Consequently, this introduces noise and variations into the analysis and contributes to reducing the model’s accuracy. Interestingly, in a sentence-based finger input context, excluding the magnetometer measurements reduces the model’s accuracy to 0.6953, underscoring its importance in capturing valuable data in handwriting dynamics.

The recognition of written words shows a similar trend, where the model reaches its highest accuracy for stylus input of 0.9576 when all sensors are active. This outcome reaffirms the importance of sensor fusion, this time for word recognition tasks. In comparison, the model based on finger input achieves its best performance (0.9286) when the readings from the piezo sensor are excluded. As the same observation also applies to finger-based signatures, we can assume that the piezo sensor introduces noise. This could potentially reduce its effectiveness in capturing the tactile details of finger movements and, consequently, lead to inconsistencies in finger-based word recognition. Similarly to sentence writing, excluding the magnetometer or smartwatch sensors (accelerometer and gyroscope) notably reduces the model’s accuracies, particularly for finger input, highlighting their importance in word classification tasks.

Letter recognition shows a slightly different pattern, with the model reaching its highest accuracy for stylus input (0.813) when all sensors are included. Here, the fusion of sensors also demonstrates its benefits, indicating that detailed multi-sensor data are essential for interpreting finer handwriting details. In contrast, the finger input-based model achieves its peak accuracy (0.6447) when the visual tracking sensor is excluded. These findings are expected, as the ArUco marker was only utilized during stylus writing to detect tilt, whereas this was not the case for finger input. Consequently, missing samples in such a scenario can introduce noise and inconsistencies in the analysis. Notably, accuracies for letter classification remain relatively lower across most sensor subsets, indicating that letters are inherently more challenging for accurate classification. This is possibly due to the very subtle differences when writing certain letters, the short time of writing, and the similarity of shapes used in forming certain letters.

Overall, the obtained results suggest that the best sensor fusion subset is highly dependent on both the handwriting form and the input modality. When all sensors are included, the model’s accuracy improves for recognizing sentences, words, and letters that are entered using a stylus. This highlights the benefit of utilizing a comprehensive multi-sensor setup. Hence, it can be concluded that including additional sensors can certainly enhance the accuracy of the model, thereby justifying their integration into the experiment apparatus.

## 5. Conclusions

In a controlled experiment focused on the acquisition of biometric characteristics of touchscreen handwriting, a total of 60 participants were involved. As opposed to similar studies which usually involve only one sensor or specifically designed electronic device, our approach utilizes an apparatus able to collect data based on the sensor fusion concept. Additionally, rather than concentrating on verification like most related studies, this research centers on identifying individuals through various forms of handwriting. Besides signatures, the focus was also on gathering handwritten sentences, words, and individual letters. The goal was to investigate alternative methods for person recognition beyond relying solely on signatures, typically considered pre-learned actions. Signatures may be susceptible to forgery, especially when skilled attackers practice replicating them. They are also commonly found on paper or scanned documents, making them easily accessible to an attacker. In contrast, longer handwriting samples, like sentences, are less available and more difficult to obtain. This supports using various handwriting types instead of signatures, as analyzing different forms of handwriting provides greater insight into a person’s dynamic writing characteristics. Employing these as an alternative “signature pattern” would make forgery attempts more challenging, requiring notably more effort to create a convincing imitation. The research was therefore extended to explore the possibilities of identification by conventional handwriting. The outcome of the conducted experiment is an original dataset comprising readings from various sensors (camera, accelerometer, gyroscope, magnetometer, touchscreen, and two piezo sensors), obtained during both stylus and finger inputs.

To facilitate handwriting recognition, a CNN-based model was developed. The accuracy of the model was analyzed with regard to the influence of three different factors: train set size, input modality, and handwriting form. During the model training process, train set sizes of 10, 20, and 30 were utilized. The obtained results demonstrate a statistically significant effect of set size on the model’s accuracy, with the highest accuracies achieved with the largest train set size.

The statistical analysis also revealed a significant difference in the model’s accuracy when writing with a stylus compared to a finger. In line with [86], finger-writing prioritizes large but less accurate motions, while stylus usage enhances precision and ensures consistency. Following the outcomes of our research, higher accuracy was consistently achieved for recognizing stylus-based handwriting. Experiment participants confirmed that the stylus provides greater precision compared to the finger, offering a writing experience similar to a conventional pen-and-paper context.

Among the different handwriting forms observed, the highest accuracy was achieved for signatures (0.982). This outcome is expected, given that each signature represents a pre-learned action unique to a particular participant. However, recognition accuracy was shown to be at a very good level for other handwriting forms as well, especially for words (0.927). The model has demonstrated a statistically significant advantage in accuracy when words were entered, compared to both sentences (0.884) and letters (0.661). This is because sentences typically have inconsistent spacing and demand more time and cognitive effort to compose. Writing shorter words repetitively enhances muscle memory, reducing mental processing and planning, which enhances focus on forming words consistently. The model’s lowest accuracy, achieved for recognizing individual letters, can be attributed to the relatively small number of strokes involved in writing letters, consequently resulting in a significantly lower amount of collected sensor data.

To address the limitations of the S Pen Remote SDK and the lack of direct connectivity between the tablet and smartwatch, alternative devices could be utilized that allow access to stylus data and enable direct connections. This would also reduce increased battery consumption from using the smartphone’s camera for visual tracking purposes.

Future work may focus on the consistency of biometric features by conducting a longitudinal study to investigate how an individual’s handwriting characteristics evolve over time. Such research would examine how factors such as aging, changes in writing habits, and physical conditions impact handwriting dynamics. Specifically, a study would involve repeated data collection from the same individuals over time to assess the evolution of biometric traits. In practical applications, production systems could overcome this challenge by continuously storing user interactions. Instead of using the data only for immediate classification, previous interactions could be archived and used for regular retraining of the model. This approach would allow the system to gradually adapt to biometric changes and maintain recognition accuracy.

From the perspective of analyzing intra-class variability and its impact on classification accuracy, our main focus was on inter-class differences in the training and evaluation of the classification model. The ability to discriminate between different individuals took precedence over within-individual variations. However, in our preliminary study, where we collected a smaller dataset from five different individuals, we performed a detailed analysis of both intra-class and inter-class variability and examined their relationship. This relationship was then formalized into a Classification Potential (CP) metric. Details regarding this can be found in the authors’ previous work [75]. A more in-depth investigation of intra-class variability, particularly the effects of changes in handwriting over time (e.g., aging-related variations), represents an interesting direction for future research.

Future studies could also explore handwriting biometrics for different languages and scripts. Collecting samples from unique character sets would help assess the universality of handwriting recognition techniques and determine if adjustments are needed for non-Latin scripts. As shown in Table 1, person recognition methods, including signature-based approaches, have been validated for several languages and scripts such as Bengali, Chinese, Uyghur, Kyrgyz, and Arabic. Although accuracy may vary, this suggests that the fundamental concept of handwriting-based recognition remains invariant to language and script. However, a dedicated experiment with a multilingual dataset could be part of future research. This would allow for a more comprehensive evaluation and provide further insight into how character structure and writing conventions affect the performance of the system.

Additionally, it is possible to investigate the effectiveness of the proposed solution when introducing new sensors or devices to the existing fusion-based apparatus. For instance, incorporating motion capture devices or electromyography (EMG) sensors could facilitate a deeper analysis of muscle activity during the handwriting process. Optical or infrared sensors can also be used to detect hand movements. These sensors may capture subtle biometric markers that could possibly improve the model’s ability to distinguish between individuals. Moreover, it is possible to experiment with different placements of piezoelectric sensors to identify the best positions for capturing pressure and vibrations on the tablet screen. Also, various models of piezoelectric sensors could be included to test their performance in this context. However, the introduction of new sensors and devices could pose several challenges, including (i) integration into the existing system, both in terms of hardware and software compatibility, (ii) adapting the data structure to the new sensor inputs, and (iii) retraining the models to leverage the additional data.

Future research directions include plans for transfer learning experiments to further contextualize our findings. Specifically, we intend to explore two key transfer scenarios: testing models pre-trained on our dataset against well-known datasets in this domain, and evaluating the performance of models pre-trained on those datasets when applied to our data. These experiments aim to provide deeper insights into the generalizability of our approach and the unique characteristics of our dataset compared to existing resources.

Finally, another aspect worth considering is the potential practical application of the proposed solution in different scenarios that, from a security point of view, either support or require handwriting-based authentication. For instance, it could be used for digital identity identification in banking and financial transactions, adding an extra layer of protection against fraud. Additionally, it can serve as an alternative biometric method for device security, such as unlocking smartphones, tablets, or secure workstations.

Expanding on this, the proposed sensor fusion-based handwriting recognition system could contribute to improving existing authentication systems in the banking sector, where transaction approvals are often signed on graphic tablets. Traditionally, these devices have only been used for digitizing signature images, but more recent implementations have begun to incorporate biometrics such as pen pressure and signing speed. Our system could potentially improve this process by integrating additional sensor data, such as the “magnetic field imprint” of the pen interaction. While this would need further testing, it could be a relatively low-cost addition that provides an additional data-based layer of security in user authentication.

Furthermore, our model could be integrated into multi-factor authentication frameworks by allowing users to enter a specific word or short phrase on a touchscreen device as an additional authentication measure. This concept is somewhat similar to TypingDNA [87], a solution that analyzes the dynamics of typing on a keyboard, but, in this case, it would be adapted for handwritten input on touchscreens.

## Figures and Tables

**Table 1:** Comparison of recent studies on handwriting-based person recognition, detailing the employed devices or sensors, handwriting forms, and the reported performance in terms of accuracy metrics.

Study	Device/Sensor	Handwriting Form	Accuracy (%)
Li et al. (2024) [48]	Image-capturing device	Handwritten signatures	98.50
Rahim et al. (2024) [53]	Tablet and a digital pen	Bengali handwriting samples, focusing on 10 distinct keywords	94.62
Leghari et al. (2024) [70]	Smartphone with a stylus	Handwritten signatures	96.00
Hasan et al. (2024) [54]	Tablet and a digital pen	10 specific keywords	98.31
Çiftçi and Tekin (2024) [49]	Image-capturing device	Handwritten signatures	98.77
Chuen et al. (2023) [63]	Microsoft Kinect camera	In-air signatures	93.00
Khoh et al. (2023) [64]	Microsoft Kinect camera	In-air signatures	97.43
Culqui-Culqui et al. (2022) [52]	Image-capturing device	Handwritten signatures	98.03
Rexit et al. (2022) [58]	Image-capturing device	Signatures in Chinese and Uyghur	92.95
Rexit et al. (2022) [57]	Image-capturing device	Handwritten signatures in Uyghur, Kazakh, and Han languages	98.40
Begum et al. (2021) [72]	Tablet and a digital pen	Defined keywords and phrases	98.00
Kette et al. [66]	Image-capturing device	Handwritten signatures	90.00
Ghosh et al. (2021) [65]	Leap motion controller	In-air signatures	94.63
Sriwathsan et al. (2021) [68]	Image-capturing device	Handwritten signatures	96.87
Poddar et al. (2020) [55]	Image-capturing device	Handwritten signatures	94.00
Akash et al. (2020) [71]	Tablet and a digital pen	Defined keywords and phrases	87.00
Pokharel et al. (2020) [50]	Tablet and a digital pen	Defined keywords and phrases	95.20
Gumusbas and Yildirim (2019) [56]	Image-capturing device	Handwritten signatures	98.80
Al-Shamaileh et al. (2019) [60]	Digital tablet device	Arabic handwriting and specific text-dependent words	81.35
Çalik et al. (2019) [51]	Image-capturing device	Handwritten signatures	98.30
Dargan et al. (2019) [61]	Image-capturing device	Handwritten Devanagari characters	91.53
Mo et al. (2019) [59]	Image-capturing device	Signatures in Kyrgyz and Uyghur	97.95
Hezil et al. (2018) [67]	Image-capturing device	Handwritten signatures	97.30
Behera et al. (2018) [62]	Leap motion sensor	Handwritten signatures	86.00

**Table 2:** Comparison of widely used handwritten signature datasets.

Dataset	Availability	Data	Format	Target
CEDAR	public	signatures	offline	offline signature verification
GPDS-960	on-request	signatures	offline	offline signature verification
MCYT-330	on-request	signatures	offline, online	offline and online verification
SVC2004	partial (training only)	signatures	online	online signature verification
SUSIG	on-request	signatures	online	online signature verification
BiosecurID	on-request	signatures, text, words, digits	offline, online	offline and online verification

**Table 3:** Impact of different sensor subsets from the proposed fusion approach on handwriting-based person recognition accuracy with a train set size of 30. The table displays accuracy scores for various handwriting forms (signatures, sentences, words, and letters) using stylus and finger inputs, with the highest scores highlighted in bold.

		Signatures		Sentences		Words		Letters	
		Stylus	Finger	Stylus	Finger	Stylus	Finger	Stylus	Finger
Excluded sensor subset	Touchscreen	**0.9911**	0.9710	0.9141	0.8884	0.9554	0.9163	0.7945	0.6347
	Magnetometer	0.9866	0.9665	0.8862	0.6953	0.9018	0.7020	0.7570	0.5019
	Input specific	0.9821	0.9821	0.8806	**0.9163**	0.9275	0.9163	0.7683	0.6344
	Piezos	**0.9911**	**0.9911**	0.9241	0.9141	0.9531	**0.9286**	0.8060	0.6293
	Smartwatch	0.9799	0.9821	0.8973	0.8683	0.9241	0.8683	0.7144	0.4973
	Visual tracking	0.9799	0.9821	0.9018	0.9107	0.9230	0.9241	0.6719	**0.6447**
	All sensor subsets included	0.9866	0.9844	**0.9263**	0.9051	**0.9576**	0.9185	**0.8130**	0.6369

## Data Availability

The data presented in this study are available on request from the corresponding author. The data are not publicly available due to privacy reasons.

## Abbreviations

The following abbreviations are used in this manuscript:

CIR	Channel Impulse Response
CNN	Convolutional Neural Network
CWT	Continuous Wavelet Transform
DTW	Dynamic Time Warping
FPS	Frames Per Second
JSON	Javascript Object Notation
LSTM	Long Short-Term Memory
MLP	Multilayer Perceptron
PPI	Pixels Per Inch
ReLU	Rectified Linear Unit
RNN	Recurrent Neural Network
SDK	Software Development Kit
SVM	Support Vector Machine
UI	User Interface
